# Rhabdomyolysis in patients with COVID-19: A cause or consequence of acute kidney injury or mortality?

**DOI:** 10.1097/MD.0000000000042368

**Published:** 2025-05-02

**Authors:** Yilmaz Mertsoy, Seyhmus Kavak, Mehmet Serdar Yildirim, Emrah Kacar, Sehmuz Kaya, Emrah Gunay

**Affiliations:** a Department of Orthopedics and Traumatology, University of Health Sciences, Gazi Yasargil Training and Research Hospital, Diyarbakir, Turkey; b Department of Radiology, University of Health Sciences, Gazi Yasargil Training and Research Hospital, Diyarbakir, Turkey; c Department of Internal Medicine, University of Health Sciences, Gazi Yasargil Training and Research Hospital, Diyarbakir, Turkey; d Department of Orthopedics and Traumatology, Van Yuzuncu Yil University, Dursun Odabas Medical Center, Van, Turkey; e Department of Nephrology, University of Health Sciences, Adana City Training and Research Hospital, Adana, Turkey.

**Keywords:** acute kidney injury, COVID-19, mortality, rhabdomyolysis, SARS-CoV-2

## Abstract

Rhabdomyolysis can occur due to many traumatic and nontraumatic causes. Rhabdomyolysis has been reported in new type of coronavirus disease (COVID-19) cases. The aim of our study was to examine the effects of rhabdomyolysis on mortality and renal outcomes in patients hospitalized in our hospital’s COVID-19 wards. In our single-center and retrospective study, we included patients who were admitted with a diagnosis of COVID-19 by a thorax-computed tomography finding who were older than 18 years of age and with a measured creatinine kinase (CK) > 1000 U/L on any day of hospitalization. The same number of patients hospitalized in COVID-19 services with CK < 1000 U/L and with similar gender and age were determined as the control group. We analyzed the data of 2065 patients, and compared 154 patients in the rhabdomyolysis group (group 1) and 154 patients in the control group (group 2). Acute kidney injury (AKI) (44.2% vs 21.4%; *P* < .001), intensive care unit (ICU) admissions (53.2% vs 13.6%; *P* < .001), intubation (75.6% vs 23.8%; *P* < .001), mortality (36.4% vs 3.2%; *P* < .001) and the need for dialysis (3.9% vs 0.6%; *P* = .005) were seen more in the rhabdomyolysis group. When that group was divided into the early rhabdomyolysis group (group 1a), where the CK value reached its highest value in ≤3 days, and the late rhabdomyolysis group (group 1b), where it was ≥ 4 days, AKI (29.7% vs 65.1%; *P* < .001), ICU (35.2% vs 79.4%; *P* < .001), intubation (56.2% vs 88%; *P* = .001), mortality (18% vs 61.9%; *P* < .001), and dialysis (1.1% vs 7.9%; *P* = .031), the results were higher in the group 1b. The available data suggest that rhabdomyolysis seen in COVID-19 patients is not a direct predictor of mortality and poor renal outcomes, but is a secondary outcome to multiple-organ failure caused by worsening clinical status.

## 1. Introduction

Rhabdomyolysis is defined as the breakdown and necrosis of muscle tissue and the release of intracellular contents into the bloodstream. It can occur due to many traumatic and nontraumatic causes. Causes such as multiple traumas, crush injuries, vascular/orthopedic surgeries and immobilization can lead to traumatic or compression-related muscle destruction. Excessive exercise, malignant hyperthermia, neuroleptic malignant syndrome, seizures, myopathies, alcohol and drug abuse, infections, some electrolyte abnormalities and endocrinopathies can be counted among the causes of nontraumatic rhabdomyolysis^.[1–5]^

Myositis due to viral infections may form a type of rhabdomyolysis. Reported cases have also been associated with influenza A/B, coxsackievirus, Epstein–Barr virus, herpes simplex virus, varicella-zoster virus, parainfluenza virus, adenovirus, echovirus, cytomegalovirus and HIV. Influenza A and B have been reported to be the most common viral myositis agents in the US.^[6]^ Rhabdomyolysis has also been reported in cases of severe acute respiratory syndrome coronavirus 2 (SARS-CoV-2) during the coronavirus pandemic.^[7–12]^ The mechanism of rhabdomyolysis due to viral agents is unclear. It has been claimed that direct viral invasion into muscle tissue, myotoxic cytokines (such as TNF-alpha) secreted in response to infection and immunological responses secondary to infection may be responsible for muscle damage.^[13,14]^

The characteristic clinical trial is muscle pain, muscle weakness and brown urine.^[1,2,15,16]^ The serum creatinine kinase (CK) level is typically very elevated. The diagnosis is confirmed when the serum CK value is >1000 U/L or at least 5 times the upper limit of normal. Electromyography (EMG), magnetic resonance imaging (MRI) and muscle biopsies are not usually required for diagnosis. However, these methods may be necessary for the diagnosis of inflammatory myopathies.

AKIN, RIFLE and KDIGO criteria were all used to determine AKI. The preferred classification criterion partially affects the determined AKI ratio. KDIGO AKI classification is more sensitive in detecting AKI. Increase in serum creatinine by ≥ 0.3 mg/dL (≥26.5 micromol/L) within 48 hours or increase in serum creatinine to ≥ 1.5 times baseline, which is known or presumed to have occurred within the prior 7 days or urine volume < 0.5 mL/kg/h for 6 hours.^[17]^ Rhabdomyolysis can be seen as an asymptomatic CK elevation, or it can be present with a life-threatening clinic. COVID-19 and other viral infections are known to cause rhabdomyolysis. Some authors claim that rhabdomyolysis increases mortality in patients diagnosed with COVID-19 and that acute kidney injury (AKI) is a strong predictor of this fatal outcome.^[18,19]^ We are not sure this hypothesis is true in patients with severe respiratory failure, such as those with COVID-19 infection. It may be overclaimed to say rhabdomyolysis is predictive of mortality in patients with severe vital organ failure. The aim of our study is to examine the effects of rhabdomyolysis on mortality and renal outcomes in patients admitted to our hospital’s COVID-19 wards.

## 2. Materials and methods

### 2.1. Participants

Our study was designed as single center and retrospective. The inclusion criteria for the study was the following: being hospitalized with a diagnosis of COVID-19 with thorax CT findings; age ≥ 18; CK > 1000 U/L on any day of hospitalization; and/or day-of-admission estimated-glomerular filtration rate (e-GFR) ≥ 30 mL/min/1.73m^2^. Since the CK cutoff value for rhabdomyolysis was accepted as 1000 in many studies, we also accepted the same limit for diagnosis.^[20,21]^ The same number of patients hospitalized in COVID-19 services with CK values < 1000 U/L, and with similar gender, age and length of stay were determined as the control group. The similarity of the same gender, age (±5 years), duration of hospitalization (±5 days) was searched for in the selection of the control group. This matching was done manually by examining the patient data. The patients who best fit the matching criteria were assigned to the control group. However, age and duration of hospitalization could not be provided for 4 of the 45 female patients in the rhabdomyolysis group. Instead of these 4 patients, 4 male patients whose similarity was provided were included in the control group.

### 2.2. Statistical analysis

SPSS Version 16.0 for Windows software (SPSS Inc, Chicago) was used to compare demographic data, laboratory data, length of hospital stay, length of stay in ICUs, rates of AKI, intubation and mortality and the need for hemodialysis. Descriptive statistics were used for demographic data. Frequency and percentages were used for categorical variables. After the normal distribution of parameters, such as age, number of hospitalization days, CK, e-GFR and CRP, were determined with the Kolmogorov–Smirnov test, they were compared with the independent sample *T* test. The chi-square test was used to compare data, including gender, intensive care admission, intubation, AKI, need for hemodialysis, intubation and mortality. A *P*-value of <.05 was considered statistically significant.

The rhabdomyolysis group was divided into 2 subgroups according to the CK-maximum value as those who reached within the first 3 days of hospitalization and those who reached after the third day, then compared.These 2 subgroups were then compared with the control group. Late occurrence of rhabdomyolysis may be related to worsening of the patient’s clinical condition and may suggest that rhabdomyolysis has an indirect rather than direct effect on renal outcomes and mortality. In the literature, we could not find a cutoff time to define early and late rhabdomyolysis groups. We assigned this limit as 72 hours.

### 2.3. Ethical approval

Approval was obtained from the ethics committee of Health Sciences University Gazi Yaşargil Training and Research Hospital (Reference number and date: 658/29.01.2021). The retrospective study design waived the requirement to obtain informed consent from patients.

## 3. Results

**Figure 1.**
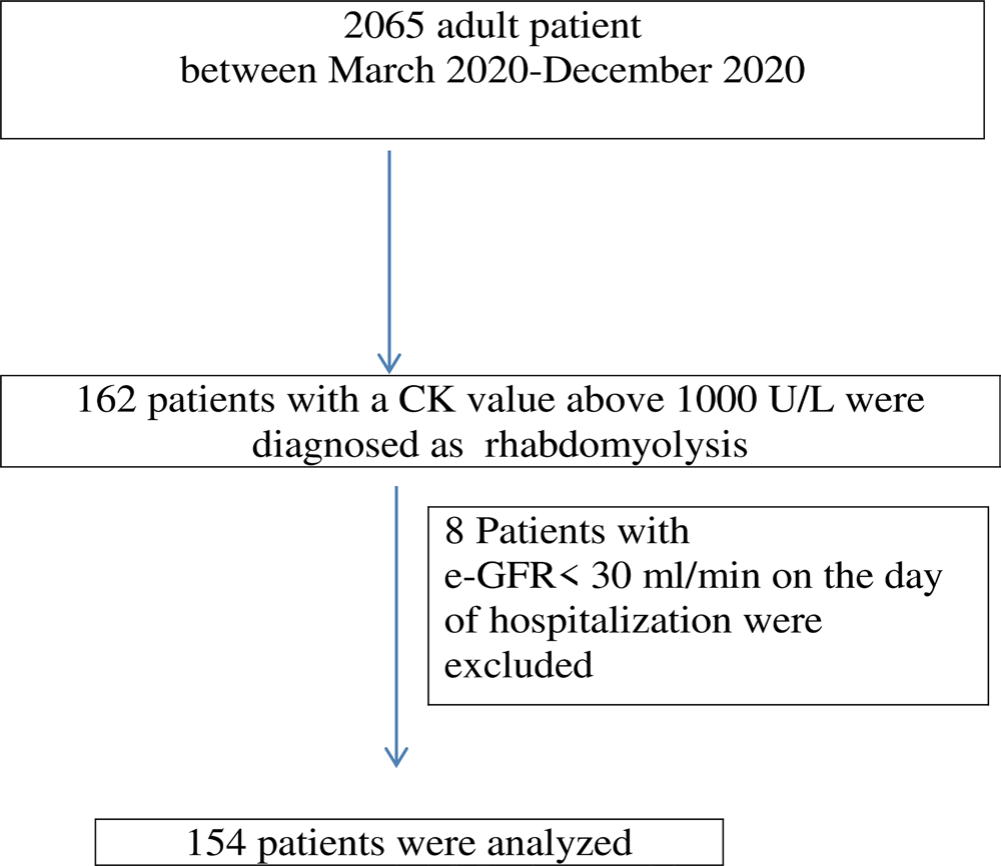
Flow chart illustrating the patient selection.

Data of 2065 patients hospitalized in our COVID-19 clinics between March 16, 2020, and December 21, 2020, were analyzed (Fig. 1). The mean age was 49.4 years (±14.4; 18–98 years), 51.4% were female and the mortality rate was 4.9%. Rhabdomyolysis was detected in 162 of the patients (7.8%). Eight patients with e-GFR < 30 mL/min/1.73 m^2^ on the day of hospitalization were excluded. A total of 154 patients in the rhabdomyolysis group (group 1) and 154 patients in the control group (group 2) were compared. Age, gender and the number of hospitalization days were similar between the groups. The following were more prevalent in the rhabdomyolysis group: AKI (44.2% vs 21.4%; *P* < .001), intensive care unit (ICU) hospitalization history (53.2% vs 13.6%; *P* < .001), intubation (75.6% vs 23.8%; *P* < .001), mortality (36.4% vs 3.2%; *P* < .001) and the need for dialysis (3.9% vs 0.6%; *P* = .0056). The e-GFR values at hospitalization were similar between the groups (75.8 ml/min vs 78.5 ml/min; *P* = .17). The e-GFR values on the last day of hospitalization were lower in the rhabdomyolysis group (55.8 mL/min vs 84.3 mL/min; *P* < .001). The data is summarized in Table 1.

**Table 1:** Rhabdomyolysis group/control group.

	Rhabdomyolysis group (group 1)	Control group (group 2)	*P*-value*	Test
Number of patients (n)	154	154		
Age (mean ± SD) (min–max)	53.99 (±16.6) (21–82)	54.12 (±16.7) (21–83)	.948	Independent sample *T* test
Female (n, %)	45 (29.2%)	41 (26.6%)	.6	Chi-square
Number of hospitalization days (mean ± SD) (min–max)	10.0 (±5.3) (3–28)	9.82 (±4.6) (3–22)	.752	Independent sample *T* test
CK-zero (IU/L, mean ± SD) (min–max)	1366 (±1920) (16–17,117)	184 (±193) (18–975)	<.001	Independent sample *T* test
CK-maximum (IU/L, mean ± SD) (min–max)	2658 (±2844) (1005–42,667)	234 (±215) (34–975)	<.001	Independent sample *T* test
CK-last day (IU/L, mean ± SD) (min–max)	913 (±3584) (19–42,667)	60 (±61) (12–304)	.003	Independent sample *T* test
e-GFR-zero (ml/min, mean ± SD) (min–max)	75.8 (±18.6) (31–90)	78.5 (±16.1) (31–90)	.17	Independent sample *T* test
e-GFR-last (ml/min, mean ± SD) (min–max)	55.8 (±34.9) (7–90)	84.3 (±12.3) (15–90)	<.001	Independent sample *T* test
Intensive care hospitalization (n, %)	82 (53.2%)	21 (13.6%)	<.001	Chi-square
Intubation (n, %)	62 (62/82, 75.6%)	5 (5/21, 23.8%)	<.001	Chi-square
Acute kidney injury (n, %)	68 (44.2%)	33 (21.4%)	<.001	Chi-square
Need for hemodialysis (n, %)	6 (3.9%)	1 (0.6%)	.056	Chi-square
Mortality (n, %)	56 (36.4%)	5 (3.2%)	<.001	Chi-square

Abbreviations: CK = Creatinine kinase, e-GFR = estimated-glomerular filtration rate, SD = Standard deviation.

* A *P*-value of <.05 was considered statistically significant for all analyses.

The rhabdomyolysis group was divided into 2 groups: the early rhabdomyolysis group (group 1a–where the CK value reached its highest value for ≤ 3 days) and the late rhabdomyolysis group (group 1b–where it was ≥ 4 days). The data for AKI (29.7% vs 65.1%; *P* < .001), ICU admission (35.2% vs 79.4%; *P* < .001), intubation (56.2% vs 88; *P* = .001), mortality (18.7% vs 61.9%; *P* < .001) and the need for dialysis (1.1% vs 77.9%; *P* = .031) were higher in the late rhabdomyolysis group (group 1b) (Table 2). When the early rhabdomyolysis group (group 1a) was compared with the control group (group 2), AKI (29.7% vs 21.4%; *P* = .14) and the hemodialysis need (1.1% vs 0.6%; *P* = .7) were similar between the groups. ICU admission (35.2% vs 13.6%; *P* < .001), intubation (56.2% vs 23.8%; *P* = .02) and mortality (18.7% vs 3.2%; *P* < .001) were worse in the early rhabdomyolysis group (group 1a) (Table 3). When we compared the late rhabdomyolysis group (group 1b) with the control group (group 2), AKI (65.1% vs 21.4%; *P* < .001), the need for hemodialysis (7.9% vs 0.6%; *P* = .03), ICU admission (79.4% vs 13.6%; *P* < .001), intubation (88% vs 23.8%; *P* < .001) and mortality (61.9% vs 3.2%; *P* < .001) were found to have worse outcomes in the late rhabdomyolysis group (group 1b) (Table 4).

**Table 2:** Early rhabdomyolysis group/late rhabdomyolysis group.

	Early rhabdomyolysis group (Group 1a)	Late rhabdomyolysis group (Group 1b)	*P*-value*	Test
Number of patients (n)	91	63		
Age (mean ± SD)	50.9 (±15.7)	58.4 (±16.9)	.006	Independent sample *T* test
Number of hospitalization days (mean ± SD)	7.7 (±4.0)	13.2 (±5.3)	<.001	Independent sample *T* test
e-GFR-zero (ml/min, mean ± SD)	80 (±15.9)	68 (±20.4)	<.001	Independent sample *T* test
e-GFR-last (ml/min, mean ± SD)	68 (±32.5)	38 (±30.6)	<.001	Independent sample *T* test
CK- maximum (IU/L, mean ± SD)	2577 (±3581)	2258 (±1000)	.63	Independent sample *T* test
Acute kidney injury (n, %)	27 (29.7%)	41 (65.1%)	<.001	Chi-square
Need for hemodialysis (n, %)	1 (1.1%)	5 (7.9%)	.031	Chi-square
Intensive care hospitalization (n, %)	32 (35.2%)	50 (79.4%)	.001	Chi-square
Intubation (n, %)	18 (18/32, 56.2%)	44 (44/50, 88%)	.001	Chi-square
Mortality (n, %)	17 (18.7%)	39 (61.9%)	<.001	Chi-square

Abbreviations: CK = Creatinine kinase, e-GFR = estimated-glomerular filtration rate, SD = Standard deviation.

* A *P*-value of <.05 was considered statistically significant for all analyses.

**Table 3:** Early rhabdomyolysis group/control group.

	Early rhabdomyolysis group (group 1a)	Control group (group 2)	*P*-value*	Test
Number of patients (n)	91	154		
Age (mean ± SD)	50.9 (±15.7)	54.1 (±16.7)	.14	Independent sample *T* test
Number of hospitalization days (mean ± SD)	7.7 (±4.0)	9.8 (±4.6)	<.001	Independent sample *T* test
e-GFR-zero (ml/min, mean ± SD)	80 (±15.5)	78 (±16.1)	.31	Independent sample *T* test
e-GFR-last (ml/min, mean ± SD)	68 (±32.5)	84 (±12.3)	<.001	Independent sample *T* test
Acute kidney injury (n, %)	27 (29.7%)	33 (21.4%)	.14	Chi-square
Need for hemodialysis (n, %)	1 (1.1%)	1 (0.6%)	.7	Chi-square
Intensive care hospitalization (n, %)	32 (35.2%)	21 (13.6%)	<.001	Chi-square
Intubation (n, %)	18 (18/32, 56.2%)	5 (5/21, 23.8%)	.02	Chi-square
Mortality (n, %)	17 (18.7%)	5 (3.2%)	<.001	Chi-square

Abbreviations: e-GFR = estimated-glomerular filtration rate, SD = Standard deviation.

* *P*-value of < .05 was considered statistically significant for all analyses.

**Table 4:** Late rhabdomyolysis group/control group.

	Late rhabdomyolysis group (group 1b)	Control group (group 2)	*P*-value*	Test
Number of patients (n)	63	154		
Age (mean ± SD)	58.4 (±16.9)	54.1 (±16.7)	.087	Independent sample *T* test
Number of hospitalization days (mean ± SD)	13.2 (±5.3)	9.8 (±4.6)	<.001	Independent sample *T* test
e-GFR-zero (ml/min, mean ± SD)	68 (±20.4)	78 (±16.1)	<.001	Independent sample *T* test
e-GFR-zero (ml/min, mean ± SD)	38 (±30.6)	84 (±12.3)	<.001	Independent sample *T* test
Acute kidney injury (n, %)	41 (65.1%)	33 (21.4%)	<.001	Chi-square
Need for hemodialysis (n, %)	5 (7.9%)	1 (0.6%)	.03	Chi-square
Intensive care hospitalization (n, %)	50 (79.4%)	21 (13.6%)	<.001	Chi-square
Intubation (n, %)	44 (44/50, 88%)	5 (5/21, 23.8%)	<.001	Chi-square
Mortality (n, %)	39 (61.9%)	5 (3.2%)	<.001	Chi-square

Abbreviations: e-GFR = estimated-glomerular filtration rate, SD = Standard deviation.

* A *P*-value of <.05 was considered statistically significant for all analyses.

## 4. Discussion

Myalgia, fatigue and malaise are associated with viral infections belonging to the coronavirus family group. A case study published in the setting of the COVID-19 infection in China identified myalgia and high CK as common findings. A CK elevation was seen in 33% of 41 patients hospitalized for Sars-CoV-2 pneumonia, and this rate increased to 46% in intensive care patients.^[22]^ In another case series of 99 patients, 11% had muscle pain and 13% had high CK.^[23]^ Muscle weakness and elevated serum CK levels were also commonly found in the 2003 SARS outbreak and the 2012 Middle East respiratory syndrome (MERS) outbreak.^[24]^ Myopathic changes, including myofibril necrosis, have been reported in postmortem histological examinations. In another study published in 2018, it was reported that viral particles were detected in macrophages-infiltrating skeletal muscles in histopathological examinations in MERS-Cove patients.^[25]^ Zhou et al reported that SARS-CoV-2 uses angiotensin converting enzyme 2 (ACE2) as a cell-entry receptor, just like SARS-Cov-2.^[26]^ During the SARS-Cove pandemic, the ACE2 receptor was described as being not only in the lungs but also in multiple-organ systems, such as skeletal muscles.

Rhabdomyolysis is usually manifested by myalgia, increased CK levels, myoglobinuria and acute renal failure. Major causes of rhabdomyolysis include autoimmune myopathies, septicemia, alcohol and drug abuse or infection. Bacterial and viral infections represent 5% of rhabdomyolysis cases, and influenza virus accounts for 42% of virus-mediated rhabdomyolysis cases.^[27]^

It is apparent in Table 1 that AKI, intensive care hospitalization, intubation and mortality data have significantly worse outcomes in the rhabdomyolysis group compared to the control group. At first glance, it may be misleading to suggest that these poor outcomes are the direct result of rhabdomyolysis. When we examined the rhabdomyolysis group in Table 2, we saw that these results were significantly different when we divided them into patients whose CK-maximum value was seen within the first 3 days of hospitalization and then declined (group 1a) and those who reached the maximum CK value after the fourth day (group 1b). We noticed that group 1b had significantly worse outcomes in AKI, the need for hemodialysis, hospitalizations in the ICU, intubation and mortality.

This picture suggests that the determinant of renal and patient survival outcomes is not the presence of rhabdomyolysis alone, but that rhabdomyolysis accompanying poor outcomes is actually a reflection of multiple-organ failure, which occurs secondary to the worsening of the clinical picture in the following days. It is more accurate to state that the main causes of poor outcomes are severe respiratory failure, multiple-organ failure, sepsis and septic shock. In addition, it does not seem reasonable to claim that the average CK values of 2658 U/L alone, as seen in the rhabdomyolysis group in our study, will lead to AKI, as it is a common complication of rhabdomyolysis. Its frequency has been reported to be between 15% and 50%.^[1,3,28]^ It is known that the risk of AKI is low in cases with a CK value of <15,000 to 20,000 U/L.^[4,28–30]^ It has also been reported that lower CK values increase the risk of AKI in the presence of dehydration, sepsis and acidosis When we compare group 1a (early rhabdomyolysis group) with group 2 (control group) in Table 3, we see that AKI and the dialysis need are similar. In other words, in patients whose CK level peaks in the first 3 days and then starts to decrease, the renal outcomes are not different from the control group.

The data also supports the above statements. When we compared the parameters of AKI and the hemodialysis need between group 1b (late rhabdomyolysis group) and group 2 (control group) in Table 4, it was understood that AKI and HD needs occurred significantly more in group 1b. In patients whose CK values were normal or close to normal at the time of their hospitalization, the reason for the CK > 1000 U/L in the following days seemed to be the worsening SARS-CoV-2 picture. Considering that the treatment approaches applied to the patients were similar, it does not seem reasonable to claim that another reason (such as drugs used, isolated electrolyte disorders) led to the development of rhabdomyolysis. From this point of view, it can be stated that rhabdomyolysis as seen in the late period reflected septic shock and multi-organ failure and is an indirect indicator of AKI and mortality.

Based on the study data, it is possible to state that the worst outcomes were in the late rhabdomyolysis group, and the best outcomes were in the control group, when we listed the data on admission to the intensive care unit, intubation, and mortality. When we evaluate the need for hemodialysis and AKI data in terms of poor outcomes, we see that the late rhabdomyolysis group had the worst outcomes. However, the early rhabdomyolysis group and the control group were statistically similar.

## 5. Conclusions

In conclusion, the available data suggest that rhabdomyolysis seen in SARS-CoV-2 patients is not alone a predictor of mortality and poor renal outcomes, but it is rather a reflection of multiple-organ failure caused by the worsening clinical condition.

### 5.1. Limitations of the study

Our study was designed retrospectively. It does not contain data on outpatients. It does not include data on vaccinated patients, as there was no vaccine developed against SARS-CoV-2 during the study period. Patient complaints for myalgia, muscle weakness and dark urine could not be reached from the file data. Patient and control group matching was done manually according to similarity profiles. Data on chronic diseases, such as diabetes mellitus, heart failure, chronic lung disease and malignancy, which may affect the results of the study, could not be obtained from the patient files. Studies including these data are needed to better evaluate renal and survival outcomes.

## Author contributions

**Conceptualization:** Yilmaz Mertsoy, Seyhmus Kavak, Mehmet Serdar Yildirim, Emrah Gunay.

**Data curation:** Yilmaz Mertsoy, Seyhmus Kavak, Sehmuz Kaya, Emrah Kacar, Emrah Gunay.

**Formal analysis:** Yilmaz Mertsoy, Seyhmus Kavak, Emrah Gunay.

**Funding acquisition:** Yilmaz Mertsoy, Seyhmus Kavak.

**Investigation:** Yilmaz Mertsoy, Seyhmus Kavak.

**Methodology:** Yilmaz Mertsoy, Seyhmus Kavak, Sehmuz Kaya, Emrah Kacar.

**Project administration:** Yilmaz Mertsoy, Seyhmus Kavak.

**Resources:** Yilmaz Mertsoy, Seyhmus Kavak.

**Software:** Yilmaz Mertsoy, Seyhmus Kavak, Mehmet Serdar Yildirim.

**Supervision:** Yilmaz Mertsoy, Seyhmus Kavak.

**Validation:** Yilmaz Mertsoy, Seyhmus Kavak, Mehmet Serdar Yildirim.

**Visualization:** Yilmaz Mertsoy.

**Writing – original draft:** Yilmaz Mertsoy, Seyhmus Kavak, Mehmet Serdar Yildirim, Sehmuz Kaya.

**Writing – review & editing:** Yilmaz Mertsoy, Seyhmus Kavak.
